# Studying longitudinal neutralising antibody levels against Equid herpesvirus 1 in experimentally infected horses using a novel pseudotype based assay

**DOI:** 10.1016/j.virusres.2023.199262

**Published:** 2023-11-17

**Authors:** Cecilia Di Genova, Gabrielle Sutton, Romain Paillot, Nigel Temperton, Stéphane Pronost, Simon D. Scott

**Affiliations:** a Viral Pseudotype Unit, Medway School of Pharmacy, Universities of Kent and Greenwich, Chatham Maritime, Kent ME4 4 TB, United Kingdom; b Animal and Plant Health Agency (APHA), Weybridge, Surrey KT15 3NB, United Kingdom; c LABÉO Frank Duncombe, 14280 Saint-Contest, France; d BIOTARGEN, Normandie Univ, UNICAEN, 14000 Caen, France; e Université de Montréal, H3C 3J7 Montreal, Quebec, Canada; f School of Equine and Veterinary Physiotherapy, Writtle University College, Writtle, Chelmsford, Essex CM1 3RR, United Kingdom

**Keywords:** Equid herpesvirus 1, Lentiviral pseudotype virus, Serology, Neutralisation assay

## Abstract

Infection with equid herpesvirus 1 (EHV-1), a DNA virus of the *Herpesviridae* family represents a significant welfare issue in horses and a great impact on the equine industry. During EHV-1 infection, entry of the virus into different cell types is complex due to the presence of twelve glycoproteins (GPs) on the viral envelope. To investigate virus entry mechanisms, specific combinations of GPs were pseudotyped onto lentiviral vectors. Pseudotyped virus (PV) particles bearing gB, gD, gH and gL were able to transduce several target cell lines (HEK293T/17, RK13, CHO-K1, FHK-Tcl3, MDCK I & II), demonstrating that these four EHV-1 glycoproteins are both essential and sufficient for cell entry. The successful generation of an EHV-1 PV permitted development of a PV neutralisation assay (PVNA). The efficacy of the PVNA was tested by measuring the level of neutralising serum antibodies from EHV-1 experimentally infected horses (*n* = 52) sampled in a longitudinal manner. The same sera were assessed using a conventional EHV-1 virus neutralisation (VN) assay, exhibiting a strong correlation (*r* = 0.82) between the two assays. Furthermore, PVs routinely require -80 °C for long term storage and a dry ice cold-chain during transport, which can impede dissemination and utilisation in other stakeholder laboratories. Consequently, lyophilisation of EHV-1 PVs was conducted to address this issue. PVs were lyophilised and pellets either reconstituted immediately or stored under various temperature conditions for different time periods. The recovery and functionality of these lyophilised PVs was compared with standard frozen aliquots in titration and neutralisation tests. Results indicated that lyophilisation could be used to stably preserve such complex herpesvirus pseudotypes, even after weeks of storage at room temperature, and that reconstituted EHV-1 PVs could be successfully employed in antibody neutralisation tests.

## Highlights

• gB, gD, gH and gL glycoproteins are required and sufficient for EHV-1 PV cell entry.
• EHV-1 specific antibodies are measurable in a pseudotype virus neutralisation assay.
• Lyophilisation can be used for stable storage and transport of EHV-1 pseudotypes.

## 1 Introduction

In the *Equidae* family, nine equid herpesviruses (EHVs) have been identified. To date, all EHVs isolated belong either to the *Alphaherpesviridae* (EHV-1, EHV-3, EHV-4, EHV-6, EHV-8 and EHV-9) or *Gammaherpesviridae* (EHV-2, EHV-5 and EHV-7) subfamilies according to the latest taxonomic classification (Davison et al., 2009; Maclachlan Dubovi and Winton Jr, 2017). Amongst EHVs, EHV-1 is considered the most virulent, as its infection is associated not only with respiratory disease but can also produce abortion, perinatal death, still-birth and neurological disorders, including Equine Herpesvirus Myeloencephalopathy (EHM) (Allen, 2002; Edington et al., 1986, 1991; Paillot et al., 2008). Thus, EHV-1 infections have a significant impact on equine welfare and lead to considerable economic losses within the horse industry as illustrated by the recognition by the USDA in 2007 of this virus as a re-emergent virus and more recently by the epizooty in Valencia (Spain) in 2021 (Couroucé et al., 2023; USDA-APHIS, 2007). Latency aids virus adaption and co-evolution with the natural host, allowing long-term survival and evasion of the immune system (Allen et al., 2004). It is estimated that the prevalence of latent EHV-1 infection is in excess of 60 % (Lunn et al., 2009). Primary infections occur in the respiratory epithelium with cell entry occurring following interaction between specific viral envelope glycoproteins (GPs) and cell receptors (Kydd et al., 1994; Patel et al., 1982).

Vaccination, in addition to good hygiene and management measures, remains an effective control practice to fight EHV-1 infection and helps reduce severity of EHV-1 related clinical manifestation (OIE, 2018). Vaccination is not mandatory, except in specific circumstances (e.g. breeding, training), and rates of uptake are difficult to estimate, but based on vaccine sales coverage appear less than 30 % in France (315 K vaccine sales for ∼1 M horses, including multiple dosing) (Nielsen et al., 2022). In addition, vaccine protection against EHV-1 disease is not always complete and cell-associated viremia has been identified in some animals, which subsequently led to EHM (Allen et al., 2004).

EHV-1 infection is routinely confirmed by Polymerase Chain Reaction (PCR) testing, detecting genomic DNA. This can be combined with virus isolation and assessment of viability of the circulating virus via cytopathic effect (CPE) (OIE, 2018). Diagnosis of EHV-1 infection is possible by serology via virus neutralisation (VN) (Thomson et al., 1976), complement fixation (CF) (Thomson et al., 1976) or enzyme-linked immunosorbent assay (ELISA) (Crabb et al., 1995) to demonstrate a virus-specific antibody response. However, due to cross-reactivity of antibodies amongst different types of EHV a type-specific diagnosis is difficult to obtain, especially between EHV-1 and −4 as a result of prior infections or vaccination (Balasuriya et al., 2015; Hartley et al., 2005). Nevertheless, serology has been extensively employed for seroprevalence surveys (Dunowska et al., 2015; El Brini et al., 2021; Gilkerson et al., 2000, 1999; Pusterla et al., 2009), and to monitor the response to vaccination (Abousenna et al., 2022; Bannai et al., 2019; Bresgen et al., 2012; Warda et al., 2021). Serology can be also used as an adjunct to inconclusive PCR results, and to confirm or exclude recent virus circulation during an outbreak situation, as recommended by the European Food Safety Authority (EFSA) (Carvelli et al., 2022). In non-vaccinated horses, EHV-1 infection can be serologically detected by screening paired serum samples collected from suspected cases during the acute and convalescent stages of infection against type-specific antigen able to demonstrate seroconversion, by a greater than 4-fold increase in antibody titre, the highest dilution of serum at which neutralisation/binding is detected (OIE, 2018). In the absence of a DIVA (differentiating infected from vaccinated animals) test, as is the case for equine influenza serology, this approach may be more complicated for herpes viruses (Galvin et al., 2013).

EHV-1 presents a complex array of twelve GPs on its surface envelope, and as observed for some other alphaherpesviruses, four GPs (gB, gD, gH and gL) are implicated as important for EHV-1 entry into cells (Azab and Osterrieder, 2012; Campadelli-Fiume G, 2007; Frampton Jr et al., 2007; Kurtz et al., 2010; Sasaki et al., 2011). More precisely, EHV-1 gB and gD are essential virus components for EHV-1 infectivity involved in virus penetration, virus release and direct cell-to-cell spread (Csellner et al., 2000; Neubauer et al., 1997). EHV-1 gH and gL, although minor components, are co-associated in a heterodimer and studies suggest their requirement in viral infection, including cell-to-cell spread (Azab et al., 2012; Harald et al., 2001).

Pseudotype viruses (PVs) offer a valuable tool to study viral entry of susceptible cells by manipulation of different combinations of candidate surface GP genes, which is more difficult to achieve with native viruses or by using reverse genetics systems. PV particles usually consist of an external envelope, displaying the GPs of the study virus, and internal core of another virus (e.g. a retrovirus) containing a modified genome, with deletions preventing virus replication. This inability to replicate allows researchers to work under low bio-containment and to focus on GP-mediated entry processes to identify the virus-cell receptor interactions and to study specific aspects of the viral binding mechanism (Temperton et al., 2015). The system can be employed to study individual GPs (e.g. haemagglutinin, HA, for influenza virus or Spike GP for coronaviruses) (Di Genova et al., 2021; Ferrara et al., 2012; Wang et al., 2004) or in combination with others (e.g. HA and neuraminidase, NA, for influenza virus) (Scott et al., 2016; Temperton et al., 2007). Consequently, this amenable PV system may also prove useful to investigate the contribution of EHV-1 GPs in cell entry. To date, no EHV-1 PV system has been established, however there is precedence within the herpesvirus family in a study by Rogalin and Heldwein (2016) in which functional herpes simplex virus (HSV-1) PV particles were generated based on a vesicular stomatitis virus (VSV) core and incorporating four different GPs. This work represented both the first herpesvirus, and indeed still the first DNA virus successfully pseudotyped. The purpose of the current study was to generate functional pseudotype particles for EHV-1, to initially investigate which of the twelve EHV-1 GPs are essential for receptor attachment and cell entry in the initial stages in virus infection. In this instance, a lentivirus core was employed, but in common with the HSV-1 PV, gB, gD, gH and gL glycoproteins were incorporated in the viral particles. In addition, as EHV-1 gC has been noted as a mediator of EHV-1 entry, driving its attachment into cells through direct envelope-plasma fusion (Csellner et al., 2000; Neubauer et al., 1997; Osterrieder, 1999), the impact of its incorporation into PV particles was examined via systemic GP substitution followed by target cell entry assessment.

PV neutralisation assays (PVNAs) offer a potential alternative to current serological tests to detect the presence of serum antibodies that can neutralise virus particles. PVNAs have been applied to serological screening, vaccine immunogenicity testing and study of the immune host response to infection by a range of different viruses (Carnell et al., 2015; Corti et al., 2011; Ferrara et al., 2015). Consequently, we utilised the functional EHV-1 PV particles generated in serological tests to measure the level of neutralising antibodies in blood serum samples which had been collected from horses over an extended time period following experimental infection with EHV-1 (Thieulent et al., 2022). The antibody titres were then compared with results obtained from VN assays using the native virus, performed using standard OIE protocols (OIE, 2018).

Transport of PVs between laboratories has been carried out typically using dry ice to maintain the cold-chain between standard storage facilities (i.e. -80℃ freezer). However, this requirement may present particular issues while shipping to warm environments (i.e. summer, the tropics) or where access to coolants are limited (as occurred during the COVID19 pandemic). Therefore, lyophilisation has been previously investigated for stable preservation of various RNA virus PVs (Mather et al., 2014; Mayora-Neto et al., 2023), but not DNA virus PV particles. Retention of EHV-1 PV titre was assessed immediately after lyophilisation and reconstitution and following one or four weeks storage under different conditions (−80℃, −20℃, +4℃ and room temperature, RT). Additionally the stability of EHV-1 PVs was measured following storage for one week at RT in polystyrene boxes surrounded with ice packs, to reflect conditions commonly used for reagent shipping. These PV samples were also tested in a PVNA, employing a small panel of EHV1-positive sera, to verify the integrity and biological function of PV to detect specific antibodies.

## 2 Materials and methods

### 2.1 Cell culture

Human Embryonic Kidney (HEK) 293T/17 cells were used for PV production, titration and neutralisation assays. Cells were grown in Dulbecco's Modified Eagle Medium (DMEM; PAN Biotech) supplemented with 10 % Foetal Bovine Serum (FBS; PAN Biotech) and 1 % Penicillin/Streptomycin (P/S; PAN Biotech) to make complete media.

Other cell lines were employed as target cells in EHV-1 PV infection experiments. Equine dermal fibroblasts (E.derm; NBL-6 ATCC® CCL-57) were maintained in complete DMEM, while rabbit kidney epithelial cells (RK13; ATCC® CCL-37) were grown in complete Minimum Essential Medium with Earle's balanced salts solution (MEM/EBSS; HyClone™, Cytiva). Chinese hamster ovary cells (CHO-K1; ATCC® CCL-61) were maintained in complete Ham's F12 medium (F-12; Gib-co™, Thermo Fisher Scientific). Foetal horse kidney cells (FHK-Tcl3) were a kind gift of Dr. Maeda (The National Institute of Infectious Diseases, Tokyo, Japan). FHK-Tcl3 and Madin-Darby canine kidney cells (MDCK I & II; ATCC® CRL-2936 and ATCC® CRL-2936 respectively) were grown in complete DMEM. All media were supplemented with 10 % FBS and 1 % P/S. All cell lines described were maintained at 37℃ in 5 % CO_2_ in a humidified incubator.

### 2.2 Gene synthesis

The full length EHV-1 gB, gD, gH and gL gene sequences (ORF 33, 72, 39 and 62 respectively) were obtained from an EHV-1 strain isolated from organs of an aborted horse foetus during a significant EHV-1 outbreak in Normandy (France) in 2010 (Sutton et al., 2019). The strain nomenclature is EHV-1 2010.203 (year and the sample ID) and belongs to the Multi Locus Sequence Typing (MLST) group 10. The GP gene sequences were aligned with the respective homologues in the reference EHV-1 strains: Ab4 (GenBank accession number: AY665713.1) (Telford et al., 1992) and strain V592 (GenBank accession number: AY464052.1) (Tearle et al., 2003) to verify the correct ORF length. All GP genes (plus upstream Kozak sequence and terminal restriction sites) were synthesised by GeneArt™ (Thermo Scientific™, Thermo Fisher Scientific) cloned into their in-house pMX plasmid vectors, with the exception of gC (ORF 16) which was synthesized and supplied as a ‘gene string’ linear DNA fragment. The EHV-1 gC sequence was obtained from EHV-1 strain Suffolk/87/2009 (GenBank accession number: KU206443.1), also belonging to the MLST group 10 (Bryant et al., 2018).

### 2.3 Plasmid preparation

gB, gD, gH and gL genes were subcloned from pMX into the pCAGGS expression plasmid (Niwa et al., 1991) previously used to produce functional pseudotypes representing a number of virus families (Di Genova et al., 2021; Kemenesi et al., 2022; King et al., 2016). The gB gene was subcloned via a blunt end strategy with *XbaI* (filled using Klenow fragment polymerase) and *XhoI* restriction enzymes and T4 ligase. The gD, gH and gL genes were cloned using *KpnI* and *XhoI* and gC gene via *EcoRI* and *BglII* restriction enzymes. All enzymes were sourced from Thermo Scientific™, Thermo Fisher Scientific. All plasmids were purified using Monarch® Plasmid Miniprep Kit (New England Biolabs) with concentration and purity determined using a Nanodrop™ 2000 Spectrophotometer (Thermo Fisher Scientific). Sanger sequencing was used to verify gene sequences (Eurofins Genomics, Germany) using customised primers based in the pCAGGS plasmid vector arms.

### 2.4 PV generation

PV generation was performed using a multi plasmid transfection system adapted from protocols used for various RNA virus families such as equine influenza (Scott et al., 2012). Briefly, HEK293T/17 were cultured in a 6-well dish the day before the DNA transfection (4 × 10^5^ cells/well). 100 µL of OptiMEM™ (Gibco™, Thermo Fisher Scientific) was mixed with plasmid DNAs: 250 ng each of the four GPs (EHV-1 gB, gD, gH and gL in pCAGGS), 750 ng of the reporter gene plasmid (pCSemGW or pCSFLW for emerald green fluorescent protein, emGFP, or firefly luciferase protein expression respectively) and 500 ng of the lentiviral HIV core plasmid (p80.91) (Sušac et al., 2022). Separately 100 µL of OptiMEM™ was mixed with 17.5 µL of polyethyleneimine (PEI; Sigma-Aldrich®) solution transfection reagent at 1 mg/mL. After 5 min RT incubation the DNA and PEI solutions were mixed, followed by a further 20 min incubation, with gentle flicking to mix. The transfection mix was then added dropwise to the wells, swirled then incubated at 37℃ for 24 h. Next, the cell culture media was substituted with 2 mL of fresh complete culture media. 48 h post-transfection, the media containing PV was collected and passed through a 0.45 µm syringe filter to remove cell debris, then stored at -80℃ until titration or next use. An additional collection at 72 h post-transfection was conducted by adding 2 mL of fresh media to the cells following the first supernatant harvest.

### 2.5 PV titration

As an initial test for successful EHV-1 PV generation, the emGFP gene was incorporated into the modified genome within the particles and entry was assessed semi-quantitatively by fluorescent microscopy on different target cells. If successful, a luciferase version of the EHV-1 PV was then produced and titre quantified. In both cases, 1:2 fold serial dilution of the PV was performed across a clear (for emGFP) or white (for luciferase) Nunc™ MicroWell™, Nunclon Delta-Treated, Flat-Bottom 96-well plate (Thermo Scientific™, Thermo Fisher Scientific); 100 µL PV in first well, then transfer across the plate into 50 µL of complete media. Next, 50 µL of target cells (1 × 10^4^ cells/mL) were added per well. A delta envelope (∆env) PV bearing no envelope GPs, plus a cell only control were included to define a threshold for successful PV production, and for cellular auto-fluorescence/luminescence background. An equine influenza (EIV) PV bearing both the haemagglutinin (HA) and neuraminidase (NA) surface GPs from the Florida clade 2 EIV strain A/equine/Richmond/1/07 (H3N8) (GenBank accession number: KF559336.1) was produced as previously reported and utilised as a positive control for the titration procedure (Kinsley et al., 2020; Scott et al., 2016). Plates were incubated for 48 h at 37℃ at 5 % CO_2_ before reading. For fluorescence evaluation, GFP-expressing cells were manually counted via a fluorescent microscope (Nikon, model: Eclipse TS100). On the other hand, Bright-Glo™ luciferase assay system (BG; Promega) was employed to measure the luminescence (in relative luminescence units; RLU per mL of supernatant) of PV supernatant. Briefly, BG was mixed with phosphate-buffered saline (PBS; PAN Biotech) in a 50:50 ratio and 25 µL/well added to the 96-well plate to wells where medium had been removed. After 5 min incubation, the plate was read on a GloMax® Navigator Microplate Luminometer (Promega).

### 2.6 Serum samples

A panel of horse serum was collected as part of an experimental EHV-1 challenge study previously described by Thieulent et al. (2022). This archived serum panel was tested for EHV-1 specific neutralising antibodies in the current study. The panel consisted of a total of 52 samples from four 10 month-old male Welsh Mountain ponies (A, B, C and D), which had been raised in a dedicated specific pathogen free facility since birth and were experimentally infected by individual nebulisation with the C_2254_ strain of EHV-1 (GenBank accession number: MT968035.1) (Sutton et al., 2020; Thieulent et al., 2022). Sample collection occurred five days before infection (A_0_, B_0_, C_0_, D_0_) and then daily from day 8 to day 18 (corresponding to sample A_8_ to A_18_, B_8_ to B_18_, C_8_ to C_18_, D_8_ to D_18_) period during which a seroconversion could be recorded. Four additional negative controls were included in the panel (E_0_, F_0_, J_0_, H_0_) (Thieulent et al., 2022). As a positive control, a multi-vaccinated pony serum was included, which had been previously used and described in EIV PV studies (Scott et al., 2012, 2016). The animal had been housed at the Animal Health Trust (Newmarket, UK) and vaccination records detail several influenza immunisations plus vaccination with the Duvaxyn® EHV 1, 4 Vaccine (Zoetis) twice in 2000. All sera were heat-inactivated at 56℃ for 30 min prior to use.

### 2.7 PV neutralisation

For PV neutralisation, sera were serially diluted in a 1:2 fold in a 96-well white plate. 1 × 10^6^ RLU of PV (previously titrated) was added to the wells. The multi-vaccinated pony serum and FBS were used as positive and negative control sera respectively. The plate was incubated for 1 hour at 37℃ to allow binding of the antibody to the antigen. Next, 1 × 10^4^ HEK293T/17 cells were added to each well. PV-only and cell-only controls were included in the plate to represent 0 % and 100 % neutralisation of the PV. The plate was incubated for 48 h at 37℃ at 5 % CO_2_ before reading. Data were normalised and plotted on a neutralisation percentage scale and the reciprocal of the serum dilution which induces 50 % neutralisation (IC_50_) was calculated using GraphPad Prism® (GraphPad) (Ferrara and Temperton, 2018).

### 2.8 PV lyophilisation and storage

In order to concentrate EHV-1 PV particles to increase usable titre, aliquots of 2 mL of both 48 h and 72 h freshly harvested PVs were low-speed centrifuged at 3000 g at +4℃ for 24 h (Jiang et al., 2015) in a bench top refrigerated microcentrifuge (VWR International Ltd, model: Micro Star 17R). Next, 1.95 mL of supernatant was then removed and discarded, making sure not to disrupt the pelleted virus, and 100 µL of cold OptiMEM™ (kept at +4℃) were added to the tube. Samples were incubated overnight at +4℃ to permit particle resuspension and stored at −80℃ before preparing samples for lyophilisation. 100 µL aliquots of EHV-1 PV supernatant were mixed with equal volume of 1 M Sucrose (Sigma-Aldrich®, Merck) solution as cryoprotectant, transferred to low retention 2 mL polypropylene microfuge tubes (Simport) and lyophilised in a freeze-dryer (FreeZone 2.5, Labconco, model: 7670560) using the method described in (Mather et al., 2014). Next, the lyophilised pellets were stored for various time periods at different temperatures: one week or four weeks at +37℃, RT with or without surrounding ice blocks (in polystyrene boxes), +4℃, −20℃ and −80℃. Lyophilised PVs were reconstituted in complete media and titrated to assess the recovery percentage.

### 2.9 Statistical tools

Raw data files produced by the luminometer were analysed using Microsoft® Excel™ 365 software (Microsoft® Windows). Column bar graphs and the non-linear regression curve fits were produced using GraphPad Prism®. Statistical analysis was performed for comparison. Normality was defined with Shapiro-Wilk test. Parametric data were analysed with a Student's *t*-test (significance at *p*<0.05) or a repeated-ANOVA test (significance at *p*<0.05). Non-parametric data were analysed with a Friedman post-hoc test (significance set at *p*<0.05).

## 3 Results

### 3.1 Generation of EHV-1 pseudotyped virus particles

**Fig. 1:**
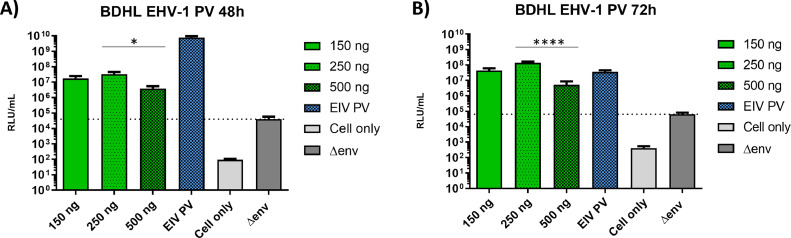
Titration of equid herpesvirus type 1 (EHV-1) pseudotyped virus (PV) particle supernatants produced with different glycoprotein-expressing plasmid combinations (gB, gD, gH and gL; BDHL). EHV-1 PV was generated by co-transfecting with either 150 ng, 250 ng or 500 ng masses of each EHV-1 GP plasmid. An equine influenza virus (EIV) PV acted as a positive control, with a no-glycoprotein PV (Δenv) and cell only as negative controls. Individual PV titres were compared to Δenv using an unpaired Student's *t*-test, all showing higher titres than this negative control (*p*<0.0001). Inter-PV titres were compared using a non-parametric Friedman test. For PVs harvested after 48 h (A), 250 ng yielded a significantly higher titre than 500 ng (* is *p* = 0.021). For PVs harvested after 72 h (B) a significant difference was also noted between 250 ng and 500 ng (*** is *p*<0.0001). Final titres (in relative luminescence units, RLU, per mL) displayed are the result of the average of duplicates in three separate experiments.

PV generation was first attempted using equal ratios of each of four candidate EHV-1 GP plasmids (gB, gD, gH, gL) testing three different masses (150, 250 or 500 ng), with GFP as a reporter for monitoring PV transduction of HEK293T/17 target cells. This provided evidence functional particles had been produced, so GFP was replaced by a firefly luciferase reporter for titre quantification assessed using PV supernatants harvested at 48 h (Fig. 1A) and 72 h (Fig. 1B) post-plasmid transfection. To confirm functionality of PVs, a Student's *t*-test was run between the PV titres and the negative control (∆env PV) which displays no surface GPs (all showed statistical significance with *p*<0.0001 in all cases). However, when comparing between the different EHV-1 PVs, a non-parametric Friedman test was employed. Interestingly, the highest plasmid amount used (500 ng) did not produce the highest titre for either the 48 h or 72 h PV harvest, with 250 ng producing the highest titre in both cases (respective statistical significance of *p* = 0.021 and *p*<0.0001). This suggests that increasing the amount of plasmid does not lead to increased titre beyond a certain threshold.. Thus, as a standard protocol to generate EHV-1 PV (and given nomenclature BDHL) 250 ng of each EHV-1 GP plasmid was employed in co-transfection and PV harvested after 72 h.

**Fig. 2:**
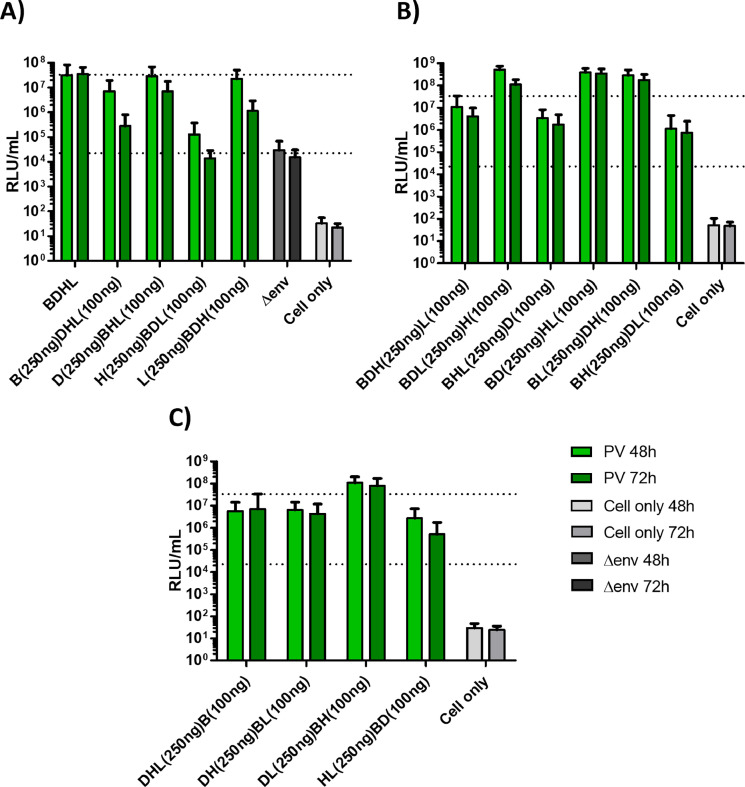
Optimisation of equid herpesvirus type 1 (EHV-1) pseudotyped virus (PV) particle supernatant titres. PVs were produced using differing amounts of co-transfected glycoprotein-expressing plasmid combinations (gB, gD, gH and gL; BDHL), and harvested after 48 & 72 h. An equine influenza virus (EIV) PV acted as a positive control, with a no-glycoprotein PV (Δenv - titre depicted as lower dotted line) and cell only as negative controls. PV titres were compared with BDHL EHV-1 PV (250 ng each plasmid; indicated by upper dotted line) standard using a non-parametric Friedman test/repeat ANOVA (see Table 1) to ascertain any statistical significance. Final titres (in relative luminescence units, RLU, per mL) displayed are the result of the average of duplicates in three separate experiments.

In attempts to further optimise EHV-1 PV titres and investigate the contribution of individual GP plasmids, different ratios of the four EHV-1 GP plasmids were tested (using 250 ng or 100 ng amounts) (See Fig. 2 for titres, Table 1 for statistical analysis of pairwise comparisons). Overall, it was found that no combination performed significantly better than the standard 250 ng of each GP plasmid protocol. Fig 2A/Table 1 reveals that using 250 ng amounts of the gD plasmid enhanced titres more than the other plasmids. Fig 2B/Table 1 demonstrate that though some combinations produce higher titres than others, none is significantly better than using four plasmids at 250 ng. This standard protocol is again shown to be superior for the Fig. 2C/Table 1 combinations.

**Table 1:** Statistical analysis of pseudotype virus (PV) titres produced from different combinations of glycoprotein (GP) plasmid transfections (depicted in Fig. 2). Only those comparisons exhibiting statistically significant differences are presented (see key for tests used). For the four GP plasmids, bold type indicates 250 ng plasmid and non-bold 100 ng (see also Abbreviations). BDHL is the 250 ng of gB, gD, gH and gH standard protocol plasmid combination. For each pairwise comparison the plasmid combination giving the significantly higher titre is given first.

**Figure 2A**				**Figure 2B**				**Figure 2C**			
PV harvest time	48hr	Time	72hr	PV harvest time	48hr	Time	72hr	PV harvest time	48hr	Time	72hr
Data	Non-parametric	Data	Non-parametric	Data	Non-parametric	Data	Non-parametric	Data	Non-parametric	Data	Parametric
Test	Friedman	Test	Friedman	Test	Friedman	Test	Friedman	Test	Friedman	Test	Rep-ANOVA
Friedman	<0.001	Friedman	<0.001	Friedman	<0.001	Friedman	<0.001	Friedman	<0.001	Rep-ANOVA	<0.001
**Pairwise comparison**				**Pairwise comparison**				**Pairwise comparison**			
**BDHL** v **B**DHL	0.044	**BDHL** v **B**DHL	<0.0001	**BDL**H v **BDH**L	0.004	**BD**HL v **BDH**L	0.001	**BDHL** v **HL**BD	0.016	**BDHL** v **DHL**B	0.035
**BDHL** v **H**BDL	0.005	**BDHL** v **H**BDL	0.001	**BDL**H v **BHL**D	<0.0001	**BDL**H v **BH**DL	0.011	**DL**BH v **DHL**B	0.005	**BDHL** v **HL**BD	0.002
**D**BHL v **B**DHL	0.027	**BDHL** v **L**BDH	0.02	**BDL**H v **BH**DL	<0.0001	**BD**HL v **BHL**D	<0.0001	**DL**BH v **DH**BL	0.027	**DL**BH and **HL**BD	0.001
**D**BHL v **H**BDL	0.003	**D**BHL v **B**DHL	0.027	**BD**HL v **BHL**D	0.002	**BL**DH v **BHL**D	0.011	**DL**BH v **HL**BD	<0.0001	**DL**BH v **DHL**B	0.008
				**BD**HL v **BH**DL	<0.0001	**BL**DH v **BH**DL	0.001			**DL**BH v **DH**BL	0.006
				**BL**DH v **BH**DL	0.017	**BD**HL v **BH**DL	<0.0001				
**Abbreviations**				**Abbreviations**				**Abbreviations**			
**BDHL** (**250 each**), **B**DHL (**B250**/DHL100), **H**BDL (**H250**/BDL100) **D**BHL (**D250**/BHL100), **L**BDH (**L250**/BDH100).				**BDHL** (**250 each**), **BDL**H (**BDL250**/H100), **BHL**D (**BHL250**/D100), **BH**DL (**BH250**/DL100), **BD**HL (**BD250**/HL100), **BL**DH (**BL250**/DH100), **BDH**L (**BDH250**/L100).				**BDHL** (**250 each**), **HL**BD (**HL250**/BD100), **DL**BH (**DL250**/BH100), **DHL**B (**DHL25**0/B100), **DH**BL (**DH250**/BL100).			

Data: data distribution tested with Shapiro-Wilk.

Friedman: Friedman *post-hoc*.

Rep-ANOVA: repeated measure ANOVA *post-hoc*.

Further investigation was undertaken to test the contribution of gC to cell entry of EHV-1 PV, by either adding gC plasmid to the BDHL combination or replacing other GP plasmids with gC plasmid in turn in transfection mixes. Incorporating gC into PVs almost eliminated a functional PV titre, and certainly did not enhance EHV-1 PV transduction of target cells. Moreover, no viable PV particles were detected when the gC plasmid serially replaced the B, D, H and L plasmids, demonstrating that the gB, gD, gH and gL combination is required for EHV-1 PV cell entry. This was confirmed by withdrawing plasmids from this standard transfection protocol, where any triple combination exhibited no target cell transduction (Supplementary Fig. 1).

### 3.2 PV transduction of different target cells

The ability of the EHV-1 PV to transduce different cell lines was assessed to identify the optimal target cell line for downstream application. An EIV PV and ∆env PV were included as positive and negative controls respectively. Relative cell transduction with PVs carrying a GFP reporter gene were conducted via fluorescent microscopy (Supplementary Fig. 2). RK13 and E.derm cells are routinely used for EHV-1 studies using the native virus (Frampton Jr et al., 2005; Peterson and Goyal, 1988), however EHV-1 PV was not able to transduce those cell lines with the same efficiency as HEK293T/17. The same was seen for the FHK cell line despite its equine origin. Further testing was conducted on BHK, CHO-K1, MDCK I and II cells, which have been employed as target cells with other PV types (e.g. Influenza, Ebola). Nevertheless, our study shows that HEK293T/17 are the cells most efficiently transduced by EHV-1 PVs of the lines tested, in addition to their role as a producer line.

### 3.3 PV neutralisation

**Fig. 3:**
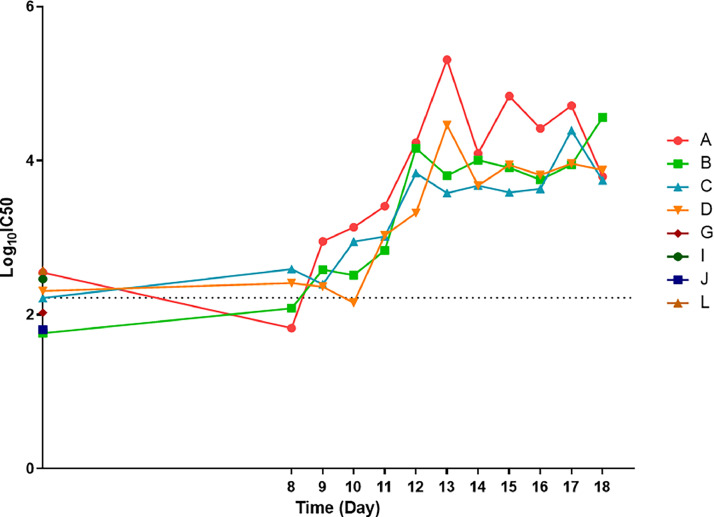
Results of equid herpesvirus type 1 (EHV-1) neutralisation assays (PVNA) of an equine serum panel. Longitudinal neutralisation patterns for each individual horse (A, B, C and D) from day 0 to day 18 (experimental EHV-1 infection occurred on Day 5) are shown. E, F G and H were sera collected at day 0 representing the four extra negative samples used to extrapolate the cut-off value (set at Log_10_-IC_50_=2.2) for detection of neutralisation. The Log_10_-IC_50_ is reported at each data point represented by the mean and standard error (IC_50_ being the reciprocal of the serum dilution which induces 50 % neutralisation).

EHV-1 PV was tested in a PVNA to assess its feasibility to be used as a serological antigen. The EHV-1 PVNA was carried out using longitudinally collected samples of horse sera following experimental infection, as described above in Section 2.6 and 2.7. Each assay included a positive and negative control to verify the test. Neat sera were added (in duplicate) at a starting dilution of 1/40 in the first well and the assay was repeated twice to verify reproducibility (Fig. 3). The 50 % inhibitory concentration IC_50_ is the reciprocal of the serum dilution which induces 50 % PV neutralisation. The threshold of positivity corresponding to an IC_50_ value of 160 (LogIC_50_=2.2) was defined by taking the average of results obtained from samples collected from all eight horses prior to the experimental infection (A_0_, B_0_, C_0_, D_0_, E_0_, F_0_, J_0_, H_0_). The antibody response increased steadily 8–10 days after infection, reaching a plateau thereafter.

### 3.4 Correlation of AB titres

**Fig. 4:**
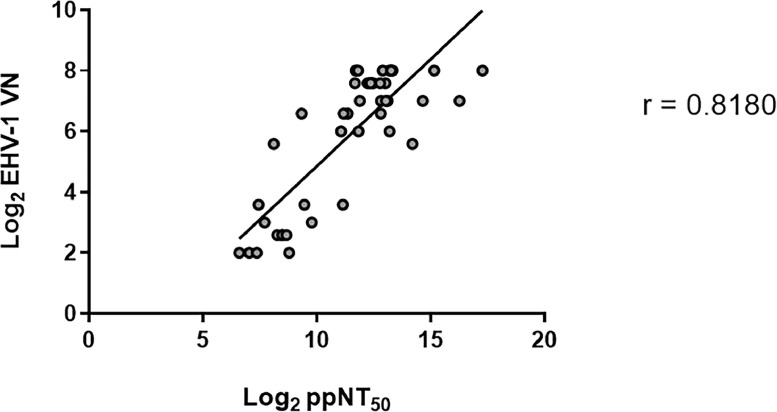
Comparison of equid herpesvirus type 1 (EHV-1) virus neutralisation (VN) assay and pseudotype virus neutralisation assay (PVNA) results. Correlation of the antibody titres obtained between the conventional EHV-1 VN (OIE 2018 protocol, with end-point titre being the serum dilution producing complete virus neutralisation) and the IC_50_ values achieved via pseudotype particle neutralisation test (ppNT_50_/ IC_50_ titre being the reciprocal of the serum dilution which induces 50 % neutralisation) are depicted as a scatter plot (log_2_ scales). The Pearson correlation coefficient *r* = 0.82 was calculated for samples collected from day 8 to day 18 (*n* = 44).

Once PVNA was successfully performed, it was deemed important to correlate the antibody titres obtained with that from a conventional EHV-1 VN assay (Fig. 4 and Supplementary Table 1). The EHV-1 VN assay was performed using the EHV-1 Kentucky D (KyD) strain (ATCC® VR-700™) and RK13 cells (Thieulent et al., 2022). Pearson correlation was calculated for all samples collected from day 8 to day 18 (*n* = 44) between the EHV-1 VN titres and the reciprocal PVNA IC_50_ values and revealed a strong positive correlation coefficient value between the two assays (*r* = 0.82, *p*<0.0001; Fig. 4) (Prion and Haerling, 2014).

### 3.5 Lyophilised PV titre

**Fig. 5:**
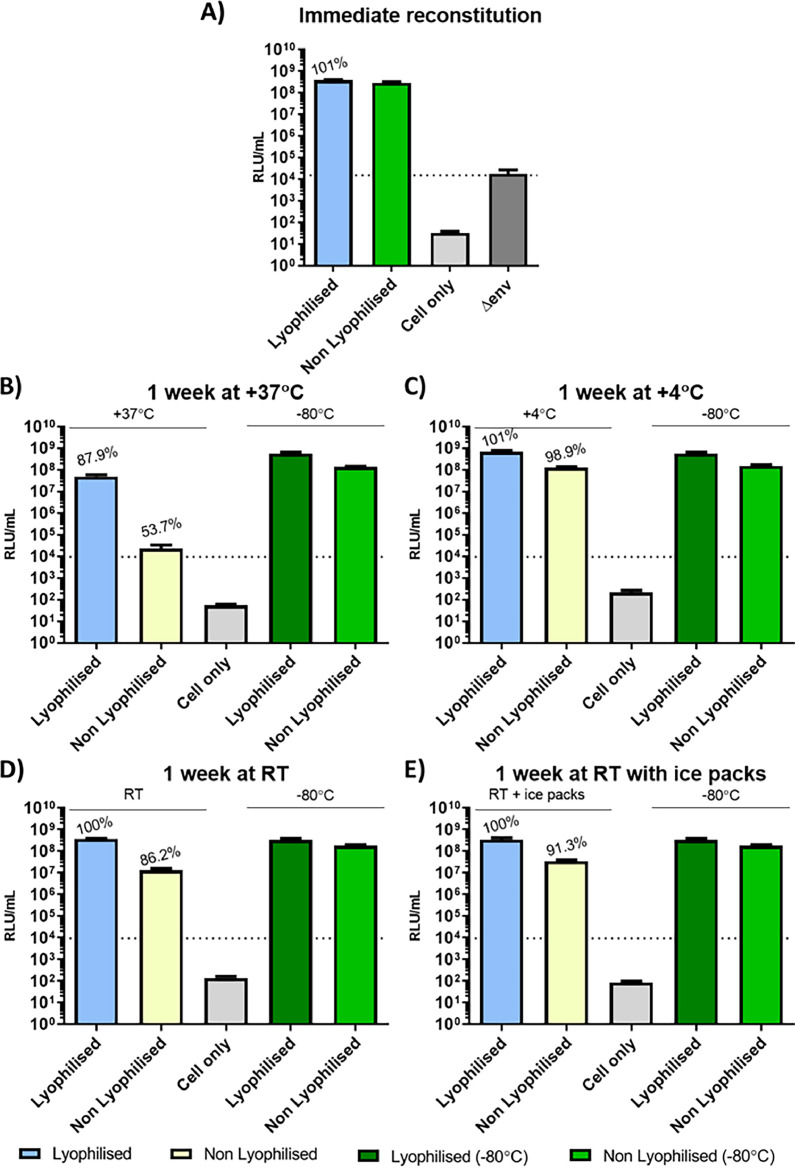
Impact of lyophilisation and storage (1 week) on titre of equid herpesvirus type 1 (EHV-1) pseudotyped virus (PV) particles. PV titre retention is reported on top of each column bar as a percentage of the absolute titre of the corresponding green column bar (−80oC storage). No glycoprotein (Δenv) PV and cell only negative controls were included to determine the cut-off value (set at 1.54×10^4^ RLU/mL and depicted as a dotted line in graphs B to E) and cell background respectively. (A) Titration results of EHV-1 PV immediately reconstituted after lyophilisation. (B) 1 week storage at +37℃. (C) 1 week storage at +4℃. (D) and (E) 1 week storage at RT without icepacks (D) and with icepacks (E) to mimic usual shipment conditions. The final titre was the result of the average of duplicates repeated once.

**Fig. 6:**
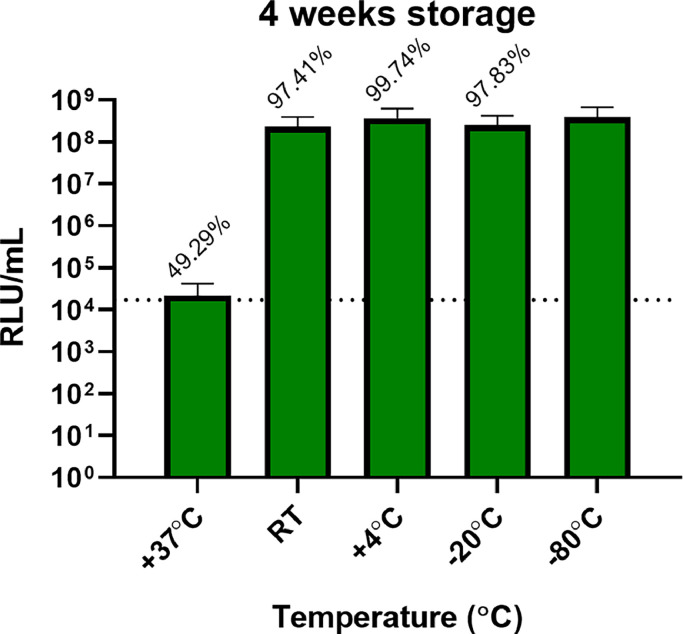
Impact of 4 weeks storage on lyophilised equid herpesvirus type 1 (EHV-1) pseudotyped virus particles at various temperatures. The% retention value above each column is a comparison with lyophilised and reconstituted PV kept at −80℃. No glycoprotein (Δenv) PV negative control is depicted as a dotted line to determine the cut-off value (set at 1.72×10^4^ RLU/mL). The final titre was the result of the average of duplicates repeated once.

To simplify shipping to stakeholder laboratories, and for subsequent long term stable storage, lyophilisation was employed as a method for preservation of PV supernatants. Following reconstitution, the retention of biological function was assessed at several time points: immediately, and one or four weeks post storage under different conditions (+37℃, RT with or without adjacent ice blocks, +4℃, −20℃ and −80℃). Percentage retention of titre was then compared with non-lyophilised control samples stored in standard −80℃ freezer. Firstly, the lyophilisation process did not to lead to significant loss of PV function following immediate reconstitution and titration (Fig. 5A). However, storage did impact retention values especially at high temperatures. After one week at +37℃ a significant loss of titre was observed (retention losses of 13 %; Fig. 5B), while lower storage temperatures reduced the impact (Fig. 5C, D and E). Importantly, storage of lyophilised PVs at low temperatures (−80℃ up to RT) for four weeks show no significance loss in titre while PV particles subjected to +37℃ lost all detectable ability to transduce susceptible target cells (Fig. 6)

### 3.6 Use of lyophilised PVs in antibody neutralisation assays

Having determined that the lyophilisation process did not negatively impact PV titres, the antigenicity of PV particles was evaluated in antibody neutralisation assays, employing a subset (*n* = 4) of samples of the serum panel. Some cytotoxicity was observed by light microscopy that when higher volumes of reconstituted PVs were employed due to the the sucrose cryoprotectant. Thus, lower volumes representing 10^5^ RLU (rather than 10^6^ RLU) were used in neutralisation assays. IC_50_ values were obtained in a range from 987 to 246,828 (LogIC_50_ 2.9 to 5.4) and the gradient of the neutralisation curves were not as consistent when compared to the PVNA employing non lyophilised PV as depicted above in Section 3.3 (Fig. 3). A lyophilised EIV PV was included as a positive control, as shown to be functional in PVNAs previously (Mather et al., 2014). In this case, both 10^5^ and 10^6^ RLU inputs of lyophilised EIV PV could be included in PVNAs, due to the smaller, less toxic volumes added. IC_50_ values were reported for both lyophilised EIV PV input conditions with slight though significant difference (LogIC_50_ 4.6 to 4.9, *p* = 0.0139; Supplementary Fig. 3).

## 4 Discussion

Pseudotyped viruses (PVs) have been shown to be useful and safe research tools to study many, almost exclusively, RNA viruses, from fundamental in vitro studies (e.g. cell tropism, receptor analysis), serology (e.g. antibody neutralisation assays), anti-viral screening and even as vaccine antigens themselves. Due to their safe, non-replicative nature, these study viruses have included a number which are classified as BSL-3 & 4 pathogens. PVs are usually easier to generate and manipulate than for example reverse genetic systems, particularly with regard to altering combinations or specifically mutating envelope glycoproteins (GPs) to analyse impact on infection. This amenability can be particularly valuable when studying complex viruses with multiple surface GPs. Herpesviruses have large DNA genomes with over a hundred genes and express a host of viral proteins on their surface. In order to study the array of equid herpesvirus surface glycoproteins, and with the ultimate aim of developing an effective test for the detection of infection or vaccine-mediated antibodies, we have successfully generated EHV-1 pseudotyped lentivirus particles bearing four glycoproteins gB, gD, gH and gL, permitting target cell entry. To our knowledge, there has been only a single report describing the pseudotyping of a herpesvirus, herpes simplex virus type 1 (HSV-1), using a VSV core and the homologous glycoproteins (Rogalin and Heldwein, 2016). Additionally, no lentiviral PVs have been reported bearing more than three envelope glycoproteins to date, and all derived from RNA viruses. One example is an influenza PV where HA was combined with both NA and M2, and was seen to increase pseudotype yields and infectivity for the PV (Wang et al., 2010). Another was the human Respiratory Syncytial Virus (hRSV) small hydrophobic protein (hRSV-SH) combined with the hRSV attachment glycoprotein (hRSV-G) and the hRSV fusion protein (hRSV-F) to investigate cell entry (Haid et al., 2016). However, in this study we demonstrate that it is possible to create a functional lentivirus PV by employing four different glycoproteins, in this case from EHV-1. We also show that the precise mass of co-transfected glycoprotein-encoding plasmids affects functional EHV-1 PV titre, with increasing amounts not always increasing titre. The current study reveals that the combination of gB, gD, gH and gL envelope glycoproteins alone are needed for EHV-1 PV particle entry of HEK293T target cells. These results were in accordance with Rogalin and Heldwein (2016) where HSV-1 VSV PVs were only able to enter C10 target cells when all four GP homologues were present. Furthermore, concentration of lentiviral particles by low-speed centrifugation increased titre by at least 1 log as seen in other studies (Cepko, 1997; Darling et al., 2000). Lastly, although expression levels of the four GPs on PVs was not measured, exclusion of any of these GPs abrogated cell transduction (Supplementary Fig. 1), indicating both that each envelope GP must have been exhibited on the viral particles but also they were biologically functional.

To optimise EHV-1 PV titre, different ratios of co-transfected GP plasmids were tested. Despite distinct differences in GP gene sequence length within the same expression vector backbone, the best results were achieved using the same amount (i.e. 250 ng) for all four. When examining the contribution of particular GPs it was notable that when the EHV-1 gH plasmid was added in higher amounts, the titre dropped significantly. Nevertheless, gH is known to be required for EHV-1 virion entry by complexing with gL, to regulate viral fusion by interaction with gB (Azab et al., 2013). In addition, EHV-1 gC is often mentioned as a mediator of EHV-1 entry into cells through direct envelope plasma membrane fusion (Csellner et al., 2000; Neubauer et al., 1997; Osterrieder, 1999). Consequently, we investigated the inclusion of gC in our EHV-1 PV particles, and whether it would enhance target cell entry. However, the incorporation of gC plasmid actually resulted in a significant decrease in EHV-1 PV titre. Additionally, sequential swapping of gC for another GP plasmid in the four plasmid sets (i.e. gB, gD, gH or gL) was tested. In each case, no measurable EHV-1 PV titre was obtained in target cell transduction experiments. Thus, despite the known role of gC in early steps of EHV-1 infection, by attaching to cell surface heparan sulphate-containing glycosaminoglycan receptor molecules, this glycoprotein was not found to be essential for EHV-1 PV entry of HEK293T cells. Despite EHV-1 having a tropism for epithelial and endothelial cells, its infectivity is not restricted to these cell types. Indeed, EHV-1 can enter permissive cells either through fusion of its viral envelope with the host cell membrane or through endocytosis (Frampton Jr et al., 2007). In addition, the host range of cell lines which EHV-1 is capable of infecting in vitro is much wider compared to other EHVs or to HSV (Whalley et al., 2007). We demonstrated that HEK293T cells were the most transducible with EHV-1 PVs suggesting that other minor EHV-1 GPs might be involved in entry of other cells, which could be further investigated using the PV system and different target cells.

Following optimisation, we were able to successfully generate EHV-1 PVs of sufficient titre for downstream use, specifically in antibody neutralisation tests. This involved utilising sera from horses experimentally challenged with EHV-1, sampled at various time intervals post infection. The results demonstrated expected patterns of neutralising antibody responses. Gradual increase of antibodies specifically neutralising EHV-1 PVs were observed from day 8, peaking around day 13 before stabilising in a plateau phase (Fig. 3). It was necessary to delineate a threshold to distinguish positive from negative samples. This cut-off was defined as an IC_50_ value of 160, obtained from the mean value of the negative samples (*n* = 8; samples collected on day 0). These samples were confirmed negative in native virus neutralisation (VN) assays also. This approach was necessary as naïve horses which have never been exposed to EHV-1 are rare. EHV-1 is remarkably ubiquitous in the horse population and it has been estimated that by two years of age, 80–90 % are infected (Allen, 2002). The difficulty of preventing the spread of infection to unexposed subjects is mostly due to asymptomatic horses after primo-infection or reactivation from latency (Allen et al., 2004; Paillot et al., 2008). So far, the results obtained with PVNA for EHV-1 are very promising, including when correlating the neutralising antibody titres with VN (*r* = 0.82, *p*<0.0001) (Fig. 4), noting that these EHV-1 PVs are displaying only four of the total twelve EHV-1 GPs. Despite EHV-1 genomes being highly conserved amongst strains, genetic variations have been observed (Nugent et al., 2006). The amino acid differences between the EHV-1 strain used for PV generation (2010.203) and the reference virus used for VN (KyD strain) could have potentially account in some part for the differences in neutralisation titre of different test sera. However, the difference in amino acid between the two strains are minimal. Comparison of the four glycoproteins showed only gB with two amino acid differences (N/H_15_ and N/D_976_). PVs could also provide an amenable tool to investigate the roles of these GPs in various combinations. For instance, it has been demonstrated that gG enables differentiation between antibodies present in polyclonal sera from mixed cases of infection involving both EHV-1 and EHV-4, by eliciting a type-specific serological response to EHV-4 (Crabb et al., 1995, 1992; Crabb and Studdert, 1993). Existing assays have shown a strong cross-reactivity in polyclonal sera due to the close antigenic similarity between EHV-1 and EHV-4 (Allen et al., 2004). Inclusion of EHV-1 gG in PV particles may be an avenue worth pursuing to develop a more type-specific antibody test. EHV-1 gG is also highly immunogenic, thus incorporating it in the PV system could give a better representation of the neutralising antibodies in sample sera.

Lyophilisation of PVs was investigated as an alternative to dry-ice shipments (and associated costs), potential customs delays, and for downstream storage. Thus, a stability study was conducted on lyophilised PVs, exposing samples to varying temperatures for various time periods, to reflect shipping conditions and subsequent short-term storage. Firstly, PV titre retention following reconstitution of lyophilised PV pellets was assessed, secondly performance in PVNAs was measured, testing different aspects of biological functionality dependant of GP integrity. Sucrose was employed as cryoprotectant as we have previously shown this to be an effective excipient (Mather et al., 2014). Stability of lyophilised PV was also assessed by measuring the titre after immediate reconstitution of PV pellets, or one-week (short-term) storage to mimic a shipment time frame scenario and after four weeks to reflect possible shipping delays, after exposure to different temperatures (Fig. 5). Excellent recovery was observed when lyophilised PV pellets were immediately reconstituted and tested (overall no significant difference between the lyophilised and non-lyophilised PVs). Following storage in different conditions, higher temperatures (+37℃) were most deleterious to functionality, as noted previously (Mather et al., 2014). Nevertheless, the lyophilised PVs were able to retain more than 87 % of their original titres respectively after one week storage at 37℃, a useful attribute if shipping at high temperatures (i.e. summer, hot countries). At lower temperatures (+4℃ and −20℃) lyophilised PVs completely retained titres compared with −80℃ storage (non lyophilised PVs dropped to 86 %). The addition of ice packs to mimic a shipping condition was able to slightly increase titre retention under RT conditions. Storage was also increased to four weeks with PVs retaining more than 97 % of their initial titres for both RT and lower temperatures (Fig. 6). By contrast, when stored at +37℃ for four weeks, no viable titre was detectable. A high functional titre is essential for the correct performance of a PVNA. Since EHV-1 PV was found to have lower titres when compared to many other PVs (e.g. influenza), the volume of reconstituted lyophilised PV which needs to be used in an assay is higher to provide a suitable amount of viral particles as antibody targets. Reconstituted, lyophilised PV samples contain cryoprotectant which can impact target cell viability. Thus, it is advisable to optimise PV titre in order to reduce input volumes and incorporate a suitable serial dilution across the assay plate.

Taking these optimisations into account, we have developed a robust and amenable system with wide utility in fundamental virological research (e.g. GP-mediated cell entry mechanisms) and an effective alternative to traditional native EHV-1 VN assays applicable to quantitative serology to investigate experimental and natural infection or vaccine efficacy.

## Ethical approval

No animal was used for this study. The archived serum panel was collected as part of the Thieulent et al. (2022) study with ethical approval and the use of archived material from this study was authorised.

## Author statement

The authors did not use any Artificial Intelligence programs/tools/services for writing, editing or data analysis at any stage in the generation of this manuscript.

## Declaration of Competing Interest

The authors declare that they have no known competing financial interests or personal relationships that could have appeared to influence the work reported in this paper.

## Data Availability

Data used in this article is avaiable on request to the authors.

## Appendix Supplementary materials

Image, application 1

Image, application 2

Image, application 3

Image, application 4

Image, application 5
