# Supplementary material for: Studying longitudinal neutralising antibody levels against Equid herpesvirus 1 in experimentally infected horses using a novel pseudotype based assay
Source: Virus Res. 2023 Nov 17;339:199262. doi: 10.1016/j.virusres.2023.199262 (PMC10694342; doi:10.1016/j.virusres.2023.199262)
Supplement: Supplementary file 1 [file mmc1.docx]

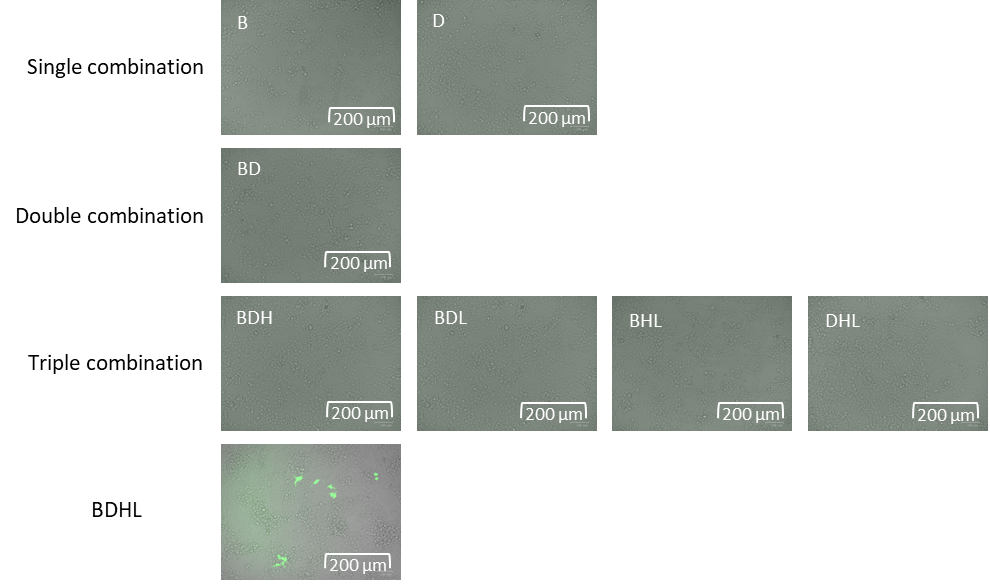


**Supplementary Fig. 1.**

Attempts to transduce HEK293T/17 target cells with pseudotype viruses (PVs) generated using plasmid expression vectors representing single, double or triple combinations of either gB, gD, gH or gL EHV-1 envelope glycoprotein (GP) genes, in comparison to the quadruple (BDHL) set. Specific GP combinations are indicated in the top left corner of each image. Cells successfully transduced by PV particles are revealed by green fluorescent protein (GFP) reporter expression – seen for BDHL only. ZOE™ Fluorescent Cell Imager (BIO-RAD) photographs are taken via a 20x objective (175x magnification), taken 48 hours post PV supernatant addition.
