# Supplementary material for: Studying longitudinal neutralising antibody levels against Equid herpesvirus 1 in experimentally infected horses using a novel pseudotype based assay
Source: Virus Res. 2023 Nov 17;339:199262. doi: 10.1016/j.virusres.2023.199262 (PMC10694342; doi:10.1016/j.virusres.2023.199262)
Supplement: Supplementary file 2 [file mmc2.docx]

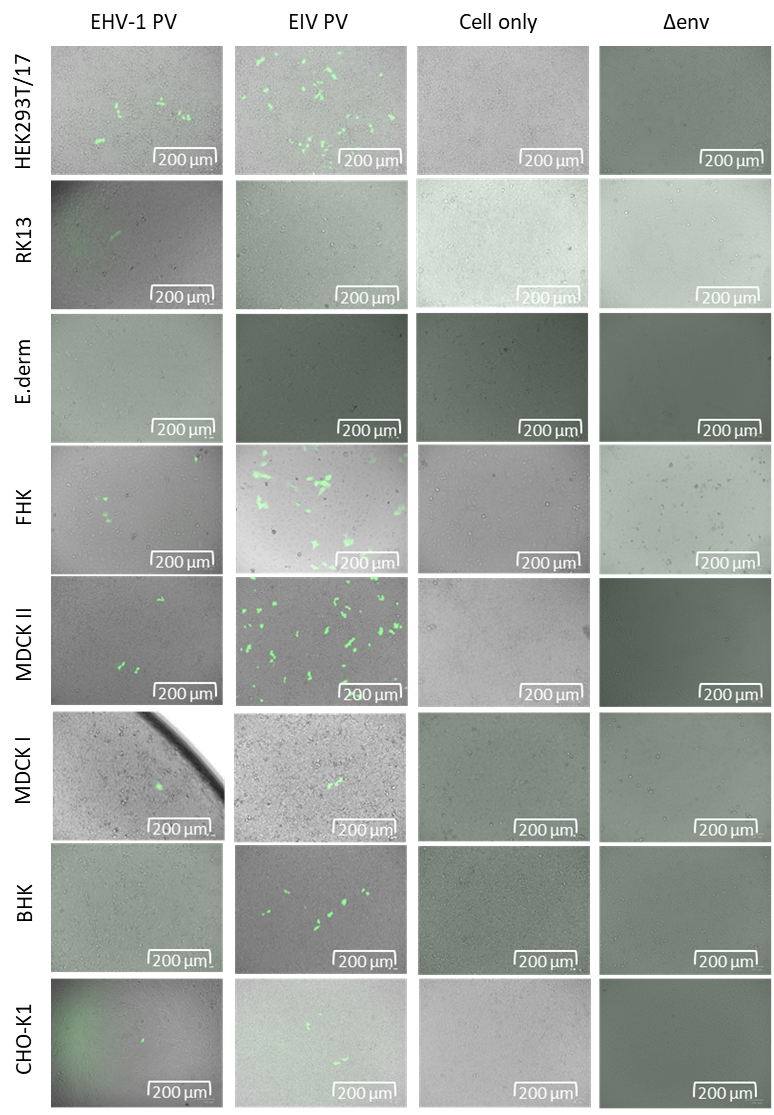


**Supplementary Fig. 2.**

Attempts to transduce various target cells (HEK293T/17, RK13, E.derm, FHK-Tcl3, MDCK I & II, BHK and CHO-K1) with pseudotype virus (PV) particles generated with gB, gD, gH or gL equine herpesvirus 1 (EHV-1) envelope glycoprotein (GP) expression plasmid constructs. Cells successfully transduced by PV particles are revealed by green fluorescent protein (GFP) reporter expression. An equine influenza virus (EIV) PV and no envelope GP (∆env) PV and cell only controls were also included. ZOE™ Fluorescent Cell Imager (BIO-RAD) photographs are taken via a 20x objective (175x magnification), taken 48 hours post PV supernatant addition.
