# Supplementary material for: Studying longitudinal neutralising antibody levels against Equid herpesvirus 1 in experimentally infected horses using a novel pseudotype based assay
Source: Virus Res. 2023 Nov 17;339:199262. doi: 10.1016/j.virusres.2023.199262 (PMC10694342; doi:10.1016/j.virusres.2023.199262)
Supplement: Supplementary file 3 [file mmc3.docx]

**
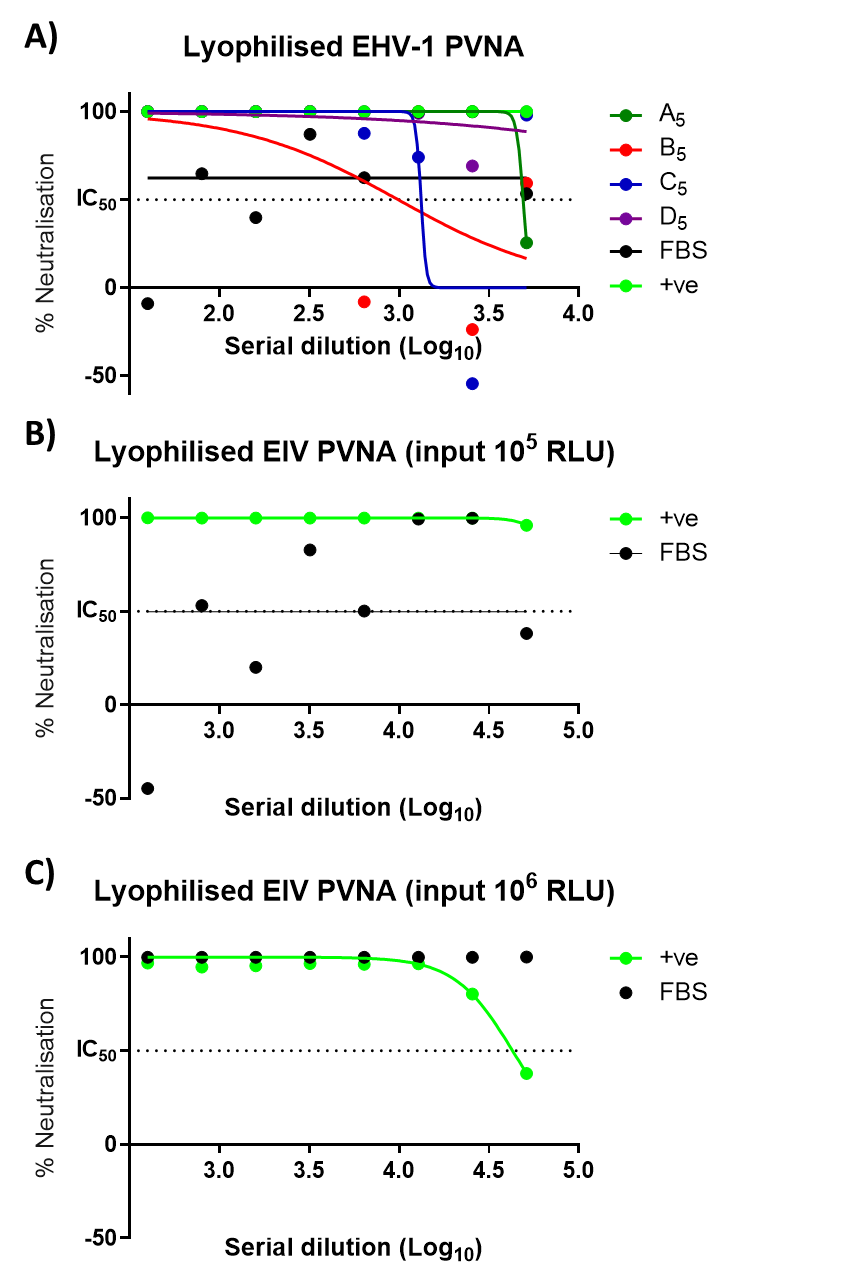
**

**Supplementary Fig. 3.**

Pseudotype Virus Neutralisation Assays (PVNA) using reconstituted lyophilised PVs and different serum samples. A) Neutralisation curves obtained testing neat sera from four horses (A-D) experimentally infected with equine herpesvirus 1 (EHV-1) tested against BDHL EHV-1 PVs (10^5^ RLU input) – details in Materials & Methods section. Serum from an EHV-1/equine influenza virus (EIV) multi-vaccinated pony (+ve) and foetal bovine serum (FBS) and were employed as positive and negative controls. B) and C) Neutralisation curves obtained with pre-diluted sera (1/10) of the multi-vaccinated pony (+ve) tested against reconstituted lyophilised EIV PVs (10^5^ RLU in B, 10^6^ in C).
