# Supplementary material for: Studying longitudinal neutralising antibody levels against Equid herpesvirus 1 in experimentally infected horses using a novel pseudotype based assay
Source: Virus Res. 2023 Nov 17;339:199262. doi: 10.1016/j.virusres.2023.199262 (PMC10694342; doi:10.1016/j.virusres.2023.199262)
Supplement: Supplementary file 4 [file mmc4.docx]

**Supplementary Fig. 1.**

Attempts to transduce HEK293T/17 target cells with pseudotype viruses (PVs) generated using plasmid expression vectors representing single, double or triple combinations of either gB, gD, gH or gL EHV-1 envelope glycoprotein (GP) genes, in comparison to the quadruple (BDHL) set. Specific GP combinations are indicated in the top left corner of each image. Cells successfully transduced by PV particles are revealed by green fluorescent protein (GFP) reporter expression – seen for BDHL only. ZOE™ Fluorescent Cell Imager (BIO-RAD) photographs are taken via a 20x objective (175x magnification), taken 48 hours post PV supernatant addition.

**Supplementary Fig. 2.**

Attempts to transduce various target cells (HEK293T/17, RK13, E.derm, FHK-Tcl3, MDCK I & II, BHK and CHO-K1) with pseudotype virus (PV) particles generated with gB, gD, gH or gL equine herpesvirus 1 (EHV-1) envelope glycoprotein (GP) expression plasmid constructs. Cells successfully transduced by PV particles are revealed by green fluorescent protein (GFP) reporter expression. An equine influenza virus (EIV) PV and no envelope GP (∆env) PV and cell only controls were also included. ZOE™ Fluorescent Cell Imager (BIO-RAD) photographs are taken via a 20x objective (175x magnification), taken 48 hours post PV supernatant addition.

**Supplementary Fig. 3.**

Pseudotype Virus Neutralisation Assays (PVNA) using reconstituted lyophilised PVs and different serum samples. A) Neutralisation curves obtained testing neat sera from four horses (A-D) experimentally infected with equine herpesvirus 1 (EHV-1) tested against BDHL EHV-1 PVs (10^5^ RLU input) – details in Materials & Methods section. Serum from an EHV-1/equine influenza virus (EIV) multi-vaccinated pony (+ve) and foetal bovine serum (FBS) and were employed as positive and negative controls. B) and C) Neutralisation curves obtained with pre-diluted sera (1/10) of the multi-vaccinated pony (+ve) tested against reconstituted lyophilised EIV PVs (10^5^ RLU in B, 10^6^ in C).

**Supplementary Table 1.** Comparison of equid herpesvirus type 1 (EHV-1) pseudotype particle test (ppNT) and native virus neutralisation (VN) assay. Antibody titres obtained by EHV-1 VN assay (using the RK-13 cell line) are compared with the reciprocal of the IC_50_ values with ppNT (log neutralisation titre) using serum samples collected from experimentally-infected (on Day 5) horses A, B, C and D, from Days 8-18.
