# Supplementary material for: Studying longitudinal neutralising antibody levels against Equid herpesvirus 1 in experimentally infected horses using a novel pseudotype based assay
Source: Virus Res. 2023 Nov 17;339:199262. doi: 10.1016/j.virusres.2023.199262 (PMC10694342; doi:10.1016/j.virusres.2023.199262)
Supplement: Supplementary file 5 [file mmc5.docx]

**Supplementary Table 1.** Comparison of equid herpesvirus type 1 (EHV-1) pseudotype particle test (ppNT) and native virus neutralisation (VN) assay Antibody titres obtained by EHV-1 VN assay (using the RK-13 cell line) are compared with the reciprocal of the IC_50_ values with ppNT (log neutralisation titre) using serum samples collected from experimentally-infected (on Day 5) horses A, B, C and D, from Days 8-18.

| **HORSE A** | | | | | | | | | | | |
| --- | --- | --- | --- | --- | --- | --- | --- | --- | --- | --- | --- |
| **Sample Day** | **8** | **9** | **10** | **11** | **12** | **13** | **14** | **15** | **16** | **17** | **18** |
| **EHV-1 VN** | 2.00 | 2.59 | 3.59 | 6.00 | 6.00 | 7.00 | 8.00 | 8.00 | 5.59 | 7.59 | 8.00 |
| **Log ppNT_50_** | 7.03 | 8.48 | 11.13 | 11.83 | 13.20 | 16.26 | 15.15 | 13.19 | 14.19 | 13.00 | 17.27 |
|  | | | | | | | | | | | |
| **HORSE B** | | | | | | | | | | | |
| **Sample Day** | **8** | **9** | **10** | **11** | **12** | **13** | **14** | **15** | **16** | **17** | **18** |
| **EHV-1 VN** | 2.59 | 3.59 | 5.59 | 6.59 | 6.59 | 8.00 | 7.59 | 7.59 | 7.59 | 7.00 | 7.59 |
| **Log ppNT_50_** | 8.26 | 7.43 | 8.10 | 9.32 | 11.35 | 11.71 | 11.67 | 12.23 | 12.47 | 14.65 | 12.32 |
|  | | | | | | | | | | | |
| **HORSE C** | | | | | | | | | | | |
| **Sample Day** | **8** | **9** | **10** | **11** | **12** | **13** | **14** | **15** | **16** | **17** | **18** |
| **EHV-1 VN** | 2.00 | 2.00 | 2.59 | 3.00 | 6.59 | 7.00 | 7.00 | 7.00 | 7.59 | 6.59 | 7.59 |
| **Log ppNT_50_** | 6.59 | 7.36 | 8.49 | 9.78 | 11.17 | 13.10 | 12.82 | 13.02 | 12.40 | 12.80 | 12.36 |
|  | | | | | | | | | | | |
| **HORSE D** | | | | | | | | | | | |
| **Sample Day** | **8** | **9** | **10** | **11** | **12** | **13** | **14** | **15** | **16** | **17** | **18** |
| **EHV-1 VN** | 3.00 | 2.00 | 2.59 | 3.59 | 6.00 | 7.00 | 8.00 | 7.59 | 8.00 | 8.00 | 8.00 |
| **Log ppNT_50_** | 7.71 | 8.78 | 8.66 | 9.45 | 11.06 | 11.89 | 11.80 | 12.79 | 12.89 | 13.31 | 13.26 |
